# Adverse Drug Events Associated with Optimizing Heart Failure Pharmacotherapy in Older Adults with Frailty: A Qualitative Study

**DOI:** 10.1016/j.cjco.2025.06.019

**Published:** 2025-07-02

**Authors:** Mai H. Duong, Danijela Gnjidic, Andrew J. McLachlan, Lisa Kouladjian O’Donnell, Ritu Trivedi, Rebecca Kozor, Sarah N. Hilmer

**Affiliations:** a Laboratory of Ageing and Pharmacology, Kolling Institute, Northern Sydney Local Health District and The University of Sydney, Sydney, New South Wales, Australia; b Northern Clinical School, Faculty of Medicine and Health, The University of Sydney, Sydney, New South Wales, Australia; c Sydney Pharmacy School, Faculty of Medicine and Health, The University of Sydney, Sydney, New South Wales, Australia; d Westmead Applied Research Centre, The University of Sydney, Sydney, New South Wales, Australia; e Cardiology Department, Royal North Shore Hospital, Northern Sydney Local Health District, Sydney, New South Wales, Australia

**Keywords:** Prescribing, deprescribing, quality of life, frail elderly, heart failure with preserved ejection fraction, adverse drug reactions, cardiology

## Abstract

**Background:**

Adverse drug events (ADEs) from heart failure (HF) pharmacotherapy are common in older people with frailty, but evidence as to how to optimize HF pharmacotherapy is unclear. This qualitative study explores consumer and healthcare professional (HCP) perspectives on ADEs and adverse drug withdrawal effects (ADWEs) related to HF pharmacotherapy to inform key domains of a conceptual model.

**Methods:**

A purposive and snowball sample of participants were contacted directly or recruited across Australia and New Zealand to participate in qualitative semistructured interviews and focus groups. Frailty was explained as a measure of cumulative deficits and consumers (caregivers or individuals aged ≥ 65 years with HF and frailty) and HCPs caring for older patients with HF and frailty were invited according to their self-perception or evaluation of frailty. General inductive analysis identified themes and a hypothesis-generating conceptual model.

**Results:**

Thirty-two participants were recruited (consumers [n = 4), cardiologists and other physicians [n = 9], nurses [n = 8], and pharmacists [n = 11]). Three main themes and 8 subthemes related to individual factors, medications, and access to healthcare services were identified. Consumers stated that they want support to maintain their quality of life but have complex medical issues. Most HCP participants perceived the benefits of HF pharmacotherapy to outweigh the risks of ADEs and are hesitant to deprescribe. Participants wanted improved coordination of multidisciplinary teams and patient access to healthcare services.

**Conclusions:**

Perspectives unique to HF pharmacotherapy in older people with frailty characterize how the interplay of HF treatment, ADEs, and ADWEs contributes to individuals’ well-being. Future research is needed to further develop the conceptual model.

## Résumé

**Contexte:**

Les effets indésirables des médicaments (EIM) entrant dans la pharmacothérapie de l’insuffisance cardiaque (IC) sont fréquents chez les personnes âgées fragiles, mais les données à l’appui de l’optimisation de la pharmacothérapie de l’IC ne sont pas suffisamment probantes. Cette étude qualitative a permis de connaître le point de vue des consommateurs et des professionnels de la santé concernant les EIM et les effets indésirables liés au sevrage médicamenteux (EISM) dans le cadre de la pharmacothérapie de l’IC, pour mieux cerner les domaines clés d’un modèle conceptuel.

**Méthodologie:**

Un échantillon raisonné, constitué en boule de neige, a été contacté directement ou recruté en Australie/Nouvelle-Zélande, en vue de participer à des entretiens semi-structurés qualitatifs et à des groupes de discussion. La fragilité a été décrite comme une évaluation de déficits cumulés, et les consommateurs (personnes fragiles âgées ≥ 65 ans présentant une IC et leurs aidants) et les professionnels de la santé qui s’occupent de patients fragiles âgés présentant une IC ont été invités à participer en fonction de leur perception ou de leur évaluation de la fragilité. Une analyse inductive générale a permis de dégager des thèmes et d’élaborer un modèle conceptuel générateur d’hypothèses.

**Résultats:**

Trente-deux participants ont été recrutés (consommateurs [n = 4]; cardiologues/autres médecins [n = 9]; personnel infirmier [n = 8] et pharmaciens [n = 11]). Trois grands thèmes et huit sous-thèmes en lien avec les facteurs individuels, les médicaments et l’accès aux services de santé ont été identifiés. Les consommateurs ont indiqué avoir besoin d’aide pour maintenir leur qualité de vie, mais qu’ils étaient confrontés à des problèmes médicaux complexes. La plupart des professionnels de la santé avaient le sentiment que les bienfaits de la pharmacothérapie de l’IC l’emportaient sur les risques associés aux EIM et hésitaient à cesser de les prescrire. Les participants souhaitent une meilleure coordination des équipes pluridisciplinaires et un meilleur accès des patients aux services de santé.

**Conclusions:**

La manière dont la pharmacothérapie de l’IC chez les personnes âgées fragiles est perçue permet de comprendre comment l’interaction entre le traitement, les EIM et les EISM influence leur bien-être. D’autres recherches s’imposent pour étoffer le modèle conceptuel.

Frailty is defined as a state of vulnerability due to reduced reserve and function of multiple physiological systems after a stressor, related to physiological aging rather than chronological aging.^1^ Frailty is determined by cumulative deficits in individuals’ physical, pharmacologic, cognitive, nutritional, psychological, and social status.^2^ Characteristics of the frailty phenotype include unintentional weight loss, weakness, exhaustion, slow walking speed. and a low level of physical activity.^3^ The prevalence of heart failure (HF) is increasing in the aging population, particularly in people aged > 75 years.^4^ Frailty is prevalent in up to 75% of older HF patients living in the community, and it is associated with an increased risk of adverse outcomes and a 2-fold greater risk of mortality.^5^^,^^6^ HF patients aged > 65 years with severe frailty have a 16% higher risk of mortality (adjusted hazard ratio [aHR] 1.16, 95% confidence interval [CI] 1.11-1.21) and a 21% higher risk of hospital readmissions (sub-distributional hazard ratio [sHR] 1.21, 95% CI 1.16-1.25) than that of robust patients.^7^

Managing symptomatic relief of HF and comorbidities can be challenging in older people with frailty. Optimization of guideline-directed medical therapies (GDMT), including contemporary and emerging treatments, can prolong life, reduce hospitalization, and improve quality of life (QoL).^8^^,^^9^ Despite the potential benefits of GDMT, patients frequently experience adverse drug events (ADEs) that impact their QoL and increase the likelihood of medication changes.^10^ Reduced tolerance to ADEs may contribute to a lower adherence level and higher rates of discontinuation in people aged > 80 years, compared to those in people aged < 65 years.^11^ As the ability to recover from injury or insult decreases, patients become more focused on improving QoL, and they report that they value maintaining their health, well-being, and happiness.^12^^,^^13^ Treatment approaches guided by medication regimens tested in trials in younger, robust populations who prioritize prolongation of life may need to be tailored for individuals with advanced age, who may value QoL optimization over longevity.^14^

Deprescribing—that is, the supervised withdrawal of a medication—may be considered in situations in which adverse effects cannot be tolerated and harm outweighs the risk of continuing medications, such as drug or disease interactions, contraindications, or acute illness. Only a paucity of evidence has been gathered to guide deprescribing or optimize HF pharmacotherapy that supports person-centred outcomes in older people with frailty.^15^ Managing polypharmacy in this population requires a multidisciplinary team to optimize therapy in line with patients’ functional status and goals.^16^ This study aims to explore the perspectives of healthcare professionals (HCPs) and consumers on ADEs and adverse drug withdrawal effects (ADWEs) of HF pharmacotherapy in older people with frailty. The secondary aims use these perspectives to inform the key domains and structure of a conceptual model for prescribing and deprescribing HF pharmacotherapy in older people with frailty.

## Methods

### Recruitment

Ethics approval for this qualitative study was obtained from the Human Research Ethics Committee at the University of Sydney (2022/811). A purposive and snowball sample was recruited from February 1 to June 15 of 2023, from across settings in Australia and New Zealand. Participants were approached directly by the researcher (M.H.D.) or recruited through advertisements distributed electronically among professional and community networks, and they were invited to contact the research team.

This study used subjective frailty assessment captured according to HCPs’ perceptions about their patients’ frailty status or participants’ self-perception of multi-system dysfunction, physical and psychosocial limitation, or evaluation of frailty. This approach aimed to recruit participants from wider geographic areas and with limited mobility. Frailty can be measured with a continuous score using a frailty index consisting of ≥ 30 factors, or the Fried phenotype, which presents frailty as 3 levels, based on the ability to predict death, falls, and functional decline.^17^^,^^18^ Objective screening tests for frailty are recommended to reduce the risk of potential underestimation from subjective assessment, but they are not applied routinely in practice. Participants were recruited until thematic saturation was achieved, once no further themes or subthemes were identified.^19^ The definition of HF was nonspecific and included undifferentiated HF, HF with reduced ejection fraction (HFrEF), and HF with preserved ejection fraction (HFpEF), the latter of which is often most prevalent among older people.

### Participants

HCPs caring for patients with HF who they identified as frail in their routine care were interviewed; these HCPs included specialist physicians (ie, cardiologists, geriatricians, hospital general physicians, advanced trainees), nurses, and pharmacists. Consumer participants included people aged ≥ 65 years with lived experience (PWLEs) of HF and frailty, or their caregivers, and consumer advocates who perceived that the person they cared for was frail. Written informed consent was obtained from all participants.

### Data collection

Semi-structured interview questions were developed with the existing frameworks for cardiovascular disease management, adverse drug reaction classifications, frailty, medication review, and the deprescribing process.^20^, ^21^, ^22^ These outcomes and adverse effects were used to develop questions pertaining to HF pharmacotherapy ADEs, particularly regarding identification, severity, cause, and management. The interview guide (Supplemental Appendix S1) was piloted with a research pharmacist (L.K.O.) and a hospital staff member aged > 70 years, for content validity. A research pharmacist (M.H.D.) with extensive qualitative methodology experience conducted interviews and focus groups either in-person or via telephone or video-conferencing with HCPs and consumers. The means of participation in interviews or focus groups was adapted to the privacy, functional capacity, and workflow needs of participants. Interviews and focus groups were audio-recorded, transcribed verbatim, verified for accuracy (by M.H.D.), and deidentified.

### Data analysis

A general inductive approach^23^ was used to guide coding of the transcripts, to explore issues related to management of ADEs and ADWEs in frail older people, to determine main themes, subthemes, and a hypothesis-generating model. Clinical outcomes, ADEs, and ADWEs were identified and classified into key domains of a conceptual model based on the main themes and subthemes. A conceptual model was derived from interpretation of the overlapping participant descriptions of the most common clinical outcomes (Supplemental Table S1), ADEs by therapeutic class (Supplemental Table S2), and ADWEs (Supplemental Table S3). Three reviewers (M.H.D., R.T., L.K.O.) independently reviewed deidentified transcripts and coded the data in Excel 2023 (Microsoft, Redmond, WA). The investigator team, which included multidisciplinary HCPs (eg, geriatricians, cardiologists, pharmacists, pharmacologists), and researchers with expertise in qualitative research, met periodically during data collection and analysis to discuss themes. Disagreements regarding themes were discussed until consensus was reached. The face validity of the themes and the conceptual model was agreed upon among the multidisciplinary investigatory team members. Data were reported according to the Consolidated Criteria for Reporting Qualitative Research checklist (Supplemental Table S4).^24^

## Results

Thirty-two participants, including 28 HCPs (specialist physicians [n = 9], nurses [n = 8], pharmacists [n = 11] and 4 consumers (PWLEs [n = 2], caregivers [n = 2]) participated in semi-structured interviews (n = 22) and focus groups (n = 4) lasting a mean of 53 ± 13.5 minutes, across a range of health settings and specialties (Table 1). Interested participants were deemed ineligible if they were PWLEs (n = 4) who described their functioning as robust and did not identify as frail, or HCPs (pharmacists, n = 3; cardiologists, n = 2; nurses, n = 2) who expressed interest in the study but did not submit consent forms after the researcher followed up. PWLEs shared that they resided in rural communities with little or no access to cardiac rehabilitation or HF support; caregivers reported having frail parents who resided in nursing homes.

**Table 1:** Participant characteristics (n = 32)

Characteristic	Value
**Consumers** (n = 4)	
Sample	
Person with lived experience Caregiver	2 (50) 2 (50)
Gender, female	3 (75)
Age, y, mean (SD)	
Person with lived experience	80 (5.5)
Caregiver	62 (0)
Average length of interviews, min, mean (SD)	52 (11.4)
**HCP** (n = 28)	
Sample	
Physician	9 (32)
Cardiologist	5 (18)
Other	
Geriatrician	3 (11)
Clinical pharmacologist	1 (4)
Nurse	8 (29)
Practitioner	4 (14)
Registered	4 (14)
Pharmacist	11 (39)
Gender, female	27 (82)
Age, y,∗ mean (SD)	45 (13.5)
Setting	
Hospital	14 (50)
Outpatient service and/or rehab and/or clinic	3 (11)
Residential aged care	1 (4)
Primary care and/or community	3 (11)
Combination	
Hospital and/or community	4 (14)
Hospital and/or cardiology clinic	2 (7)
Cardiology clinic and/or community	1 (4)
Time in practice	
1–10	7 (25)
role, y	
11–20	9 (32)
> 20	12 (43)
Average time, min, mean (SD)	
Overall	54 (24.1)
Interviews	52.1 (12.5)
Focus groups	54.9 (8.8)

Values are n (%), unless otherwise indicated.

HCP, healthcare professional; rehab, rehabilitation; SD, standard deviation.

∗ 4 HCP participants did not disclose their age.

The 3 main themes identified and supported with supporting quotes were as follows: theme 1, individual factors (Table 2); theme 2, medications (Table 3); and theme 3, access to healthcare services (Table 4). Eight subthemes describe management and characteristics of ADEs and ADWEs in this population. General and therapeutic class-specific ADEs and ADWEs are described in Supplemental Tables S2 and S3. Consensus on the identification of outcomes, ADEs, and ADWEs reported showed that all participants shared similar goals for patients. However, the approach to achieving these outcomes, and which aspects of the individual should be supported, differed among specialties. A wide range of views were expressed in response to the semi-structured questions. This range was due to complex and dynamic factors, including frequency of medications, severity of adverse events, which aspect of an individual’s QoL was impacted, knowledge of which ADEs to monitor, availability of caregiver support or access to HCPs, and rehabilitation programs.

**Table 2:** Quotes supporting subthemes and categories for theme 1—individual factors

Reference	Subthemes	Quotes
**Complex patient factors**		
Quote 1	Changes in function	In older patients, I see a lot of high potassium, renal function is off and [their] blood pressure has dropped.—pharmacist 9
Quote 2	Frailty looks at the whole patient	It's that extra effort one has to put into chores. It's quite hard. It's a range of [anxiety] about things and may come from the stroke and having low vision. I go to do something and feel like I can’t. In all fairness, I can't blame the medication for that.—consumer 55
Quote 3	Defining frailty is complex	You have to adapt your approach to frailty. I don't know how good I am, or my field is, at engaging frailty. The way I gauge frailty might be very different to someone else. I tend to overcall frailty based on mobility, which is not the same thing.—cardiologist 29
**Supporting patients**		
Quote 4	Dignity	[Diuretics] caused frequency [sic]. The thought that [dad] could humiliate himself by not making it to a bathroom in time is degrading. It's challenging to an older person's dignity, and the knock-on effects are they tend to withdraw from social activities. They don't want to risk making fools of themselves [or] be too far from a bathroom.—consumer 53
Quote 5	Goals of care	I struggle a bit. It's navigating that without actually worrying or hating all of it. All they want to know [is if] you’re getting better. Sometimes you're not feeling all good, don't feel much better. It's a tricky path we tread as we get older and with the frail.—consumer 55
Quote 6	Support of carers and advocates	The goal is to improve connections so I can be confident enough to do the things I used to thoroughly enjoy doing. I am very lucky I've got people that care. I'm in quite a privileged safe environment. I’m not alone. I've got family and good support.—consumer 55

**Table 3:** Quotes supporting subthemes and categories for theme 2—medications

Reference	Subthemes	Quotes
**Medication management**		
Quote 7	Evidence-based medicine	Quality of life is at the top. Rebuilding a joie de vivre [joy of living].—consumer 55
Quote 8	Adjustments and limitations of GDMT in frailty	Single agent might be more appropriate and up-titrating that one, rather than the combinations of medications that can increase adverse effects.—pharmacist 18
Quote 9		The titrating and the people coming in and out of their life. It's the [use of] diuretics, the pill burden, and the burden of getting to and from medical appointments in the frail that potentially have equal consequences as the tablets.—nurse 10
Quote 10	Gap in the evidence	HFrEF’s pretty straightforward as to what the evidence is. HFpEF is still evolving. I find it so much more difficult to treat.—nurse 11
Quote 11	Polypharmacy is a separate issue from prioritizing clinical outcomes	The issue of polypharmacy, pills interacting and having too many pills, is a separate issue. It's a matter of which ones [sic] required, and which are not. Patients don't like taking medications; I wouldn't, no one does. Polypharmacy is a real issue. Cardiologists get a label they struggle to stop things, pro-intervention, pro-treatment, pro-lengthening life, potentially at a cost.—cardiologist 29
**Identification and management of ADEs**		
Quote 12	Identify, report, investigate symptoms	You could have frequency and incontinence with diuretics. Dehydration with rise in creatinine, people feeling weak, dizzy and often their blood pressure goes down. Electrolyte abnormalities, like low potassium or sodium, often is symptomatic. They may be drowsy, low or reduced level of consciousness, hypotensive, and very dry. Delirium is not just dioxin toxicity. You can have delirium as a consequence of any of these side effects, low sodium, dehydration, AKI [acute kidney injury].—physician 19
Quote 13		If you don't have the skills to recognize fluid overload, you’ll miss the decompensation and treat the symptoms as being medication-related and not decompensation. Worsening creatinine and they don’t assess the volume status. Non-HF doctors will go, ‘the creatinine is rising, they’re dehydrated. Quick stop things’. That can be worsening congestion.”—cardiologist 12
Quote 14	Severity of ADEs	When withdrawing therapies because of QoL, is it the medication causing this deterioration or something deeper? Some patients are so cachexic, frail and malnourished with albumin above 15. Is ketoacidosis progression of their dementia due to effectively no oral intake or is this HF-related?—cardiologist 28
Quote 15		Rise in creatinine is so ubiquitous in cardiac failure, in that you always ignore them unless they're very impressive. Blood pressure… We’re big advocates of, ‘if it’s not symptomatic hypotension, it's not hypotension’. It's life-limiting symptoms that matter. If it's tolerable, not life-limiting or not hospitalization-inducing, then it doesn't really matter that much, you kind of ignore it or try and push through.—cardiologist 12
Quote 16	Lack of ADE patient knowledge	When you have so many medications, you don't know the effects of one medication against the other. Whether it could be a mixture of that medication that's causing a problem.—consumer 50
Quote 17		It depends on the person. A lot of patients volunteer everything and a lot volunteer nothing until you probe them. Then other people grin and bear, don't really notice the side effects, but if you ask, on next follow-up they would tell me. I always ask about postural hypotension, dizziness, palpitations, chest pain tightness, breathlessness, exercise intolerance in every patient.—cardiologist 25
Quote 18	Weigh benefits vs risks	The problem is the number of medications and potential interactions. One of our big issues is patients falling down with hypotensive drugs. With furosemide, needing to get to the bathroom quickly [is a] falls concern.—nurse 13
**ADWE considerations**		
Quote 19	Attitudes to deprescribing	Discontinued can be different than stopped for a while. Doesn't mean they can't ever have it again. It just means we need to start again, but not all of them. If they need some antibiotic that's going through the kidneys, then you are going to give that priority and stop diuretics. We can stop [SGLT2-i] and start them again. It's flexible.—nurse 6
Quote 20	Weigh risks vs benefits of deprescribing	We leave the HF meds in because we think it might be contributing to them feeling better. We’re trying to make things better and taking those drugs away may make them feel worse. It's to try and improve their QoL, reduce hospitalizations, reduce death.—pharmacist 4
Quote 21		I fully support deprescribing, particularly in older patients. But if you're committed to HF, I don't support deprescribing. Those patients have got to be on it. There's literature around stopping medications at the end-of-life.—physician 24
Quote 22	Lack of evidence on deprescribing	If they've HFpEF, [sic] it's easy. There's no evidence for it. Although it's interesting, the evidence from [SGLT2-i trials].—physician 23
Quote 23	Identification and monitoring of ADWEs	It's more than patients reporting how they feel. You have to anticipate harm to that patient, because they're not going to feel hyperkalemia until they have an arrest. There’s clear harm it’s going to cause that patient because we've initiated or up titrated their ACEI. Hyperkalemia is a likely cause of hospitalization we want to try and minimize. Sudden hypotension related to a change, if a big change rather than a small change, degrees of change [sic].—nurse 14
Quote 24		If you have to constantly cease and then restart, because they can't tolerate, I don't think that's a good idea. Especially with Entresto, when you start that's where hypotension affects [them] and then before you know [it] you have to stop again.—cardiologist 27
**Facilitators and barriers to patient education about benefits and harms**		
Quote 25	Balance medication recommendations with QoL	Getting a new or upping a medication I always talk to the GP—‘how's this going to affect everything else that I take?’ Because that's important. They throw in the tablets and say, ‘oh, this will be good for bloating,’ or something like that. But I always ask, ‘how does it affect everything else?’—consumer 55
Quote 26	Navigating evidence	These patients have very limited capacity to take on information. When you first see them, [they] can't follow conversations, [are] slow. I write everything down and give them to take it home.—physician 24
Quote 27	Communication to patients about medications and ADEs	When I'm starting someone and/or titrating an ACEI, I'll say, ’This will make you feel a bit woozy for the next three days. That means sit to stand, turn your head suddenly. It doesn't mean you can't drive, you can't do the things that you've been doing. It just means to go gently for the next couple of days. If you're lying on the couch and watching television and someone's at the front door and you jump up to answer it, you're going to fall over. So just go gently.’—nurse 6
Quote 28		I've changed doctors because some of them I just got, ’no, I don't know.’ I really struggle with them because of their ideas and then they've got no idea on medication. I don't know why, but I get more brains out of my pharmacist. He's very good.—consumer 50
Quote 29		Not that GPs don't care, [but] that it's not their focus. They have 5-15 minutes with a patient. They don't do pre-reading. They don't have the capacity. Whatever they've got in front of them, they do… [The cardiology team] see patients the most and do that. The whole goal of many of the clinics is to titrate patients up to the maximally titrated doses.—nurse 6

ACEI, angiotensin-converting enzyme inhibitor; ADE, adverse drug event; ADWE, adverse drug withdrawal event; GDMT, guideline-directed medical therapy; GP, general practitioner; HF, heart failure; HFpEJ, heart failure with preserved ejection fraction; HFrEF, heart failure with reduced ejection fraction; QoL, quality of life; SGLT2i, sodium glucose cotransporter 2 inhibitor.

**Table 4:** Quotes supporting subthemes and categories for theme 3—access to healthcare services

Reference	Subthemes	Quotes
**Integrated and interprofessional coordination**		
Quote 30	Need coordinated multidisciplinary expertise	The final common denominator for the geriatrician is overall function. Whereas for the cardiologist, it's cardiac function.” —physician 19
Quote 31		The battle is, not always but sometimes, each of the individual organ systems are actually in opposition. That's the challenge for us as a multidisciplinary team, is we're all trying to optimize the organ system that we are specialists in to the best of our ability. But it comes at a cost to other organ systems when you have a frail patient.—cardiologist 28
Quote 32		At the end of the day, if you are a clinician looking after this patient, like a geriatrician, you should make the call, don’t even bother consulting cardiology. Some teams will do too much and some too little. It’s better if there’s some middle ground that the team can get to [and] is doctor-dependent [sic]. Especially if they’re quite frail and advanced in age, probably cardiology is not going to be the best team to manage them.—cardiologist 27
Quote 33	Monitoring and follow-up	Whether it's HFrEF or HFpEF, it’s very difficult to find in medical notes and discharge summaries to see the results of an echo, which are not often enclosed. Sometimes the cardiologist is the only one who knows whether it's HFrEF or HFpEF.—pharmacist 2
Quote 34	Multiple healthcare settings	I’ve gotten very frail patients to target doses over a long time. They get hospitalized for something else and non-cardiac teams stop all their pills. I build them back up again from scratch.—cardiologist 12
Quote 35		I've got much better at deliberately involving the cardiologist in my deprescribing. I'll send an email straight away. Whereas previously, I just deprescribed. It's not good patient care ‘cause what'll happen is you'll reduce it, [the patient] will then go and see the cardiologist and restart it. And the poor GP’s caught in between.—physician 23
**Patient access to care**		
Quote 36	Challenges to accessing care	True adverse effects, the serious ones, don't come to a clinic. You get the reason why something started was stopped taken out of context, it's tricky. There's a role for HF specific clinics and nurse practitioner education clinics where someone is dedicated and patients are followed a lot more.—cardiologist 29
Quote 37		Some patients don't have GPs they can get into and/or they need a home visiting GP because they're not mobile and it's not [available]. It's really hard. Part of our role in the community is to check and make sure the GP is getting enough support. The GPs in this area are very overworked. It takes our patients a couple of weeks to get an appointment.—nurse 10
Quote 38		I haven't had any support. I tried to ring up the heart mob and see if I could go along and do exercise or whatever. But nothing's happened. They gave me a lady's name and I rang the hospital a few times and I couldn't get through. I just give up in the end. I just do it myself. I think I would've gone okay with rehabilitation. They may have pushed you more to do things. I went through the whole thing on my own. I had no support when I was out of hospital because of where I lived.—consumer 50
Quote 39	Coordination of care	[To see a cardiologist], do you have to find accommodation somewhere? Most of them don't drive. There is no public transport. People in the [rural] community if they haven't got funds and transport, they would go without an echo, even people who are not frail necessarily. They haven't got the access. We have quite a low socioeconomic population here.—pharmacist 4
Quote 40		We made an appointment to take him on the following Wednesday to start his exercise program. [But], dad died on the Sunday night. I was so sad.—consumer 53

echo, echocardiogram; GP, general practitioner; HF, heart failure; HFpEF, heart failure with preserved ejection fraction; HFrEF, heart failure with reduced ejection fraction.

### Theme 1: individual factors

#### Complex patient factors

All HCPs and consumers described that older people living with frailty and HF have multiple complex issues to consider; including their HF phenotype, changes in cognition and function, and interaction with treatment for other comorbidities (Table 2, quote 1). These considerations can influence the extent of benefit received from HF pharmacotherapy and/or the ability to tolerate adverse effects (Table 2, quote 2). Objective measures of frailty are not assessed routinely in practice, despite the benefit of defining frailty status to determine individuals’ potential reserve, ability to recover from injury or insult, and ability to adjust to functional and cognitive changes. HCPs described various approaches to considering frailty in management of patients with HF (Table 2, quote 3).

#### Supporting patients to achieve their goals of care

Participants indicated that older people with frailty want the dignity, confidence, and choice to live independently at home, perform daily activities they enjoy, and maintain social engagements. For example, individualized adjustments of diuretics were a common concern during prescribing or deprescribing. Personal autonomy may be respected by discussing co-management of incontinence, concurrent GDMT to improve HF symptoms and reduce the need for diuretics, and how to manage adverse effects (eg, incontinence, falls, dehydration, social isolation; Table 2, quote 4). Individuals’ goals and expectations can vary. Participants suggested that the support of caregivers and advocates can support shared decision-making to optimize individuals’ QoL and allow them to perform the activities that are valuable to them. Full disclosure about benefits and risks of treatments, symptom management, functional limitations, social support, and potential impacts on other health conditions can support individuals’ adherence to changes in prescribing or continuation of their current medication (Table 2, quote 5). They also acknowledged that the balance between optimization and QoL should be flexible and tailored for individuals with limited function and frailty (Table 2, quote 6).

### Theme 2: medications

#### Medication management within the context of limited evidence

Participants described different views on how they prioritize medication use and health outcomes. Consumers explained that maintaining their QoL was most important (Table 3, quote 7). HCPs described using evidence-based GDMT to prolong life, reduce hospitalization, and improve functions needed to perform activities of daily living (ADLs). HCPs described that optimizing GDMT entails dynamic and frequent changes. These changes include re-challenging or up-titrating medications once ADEs resolve, to optimize the benefit of medications to treat HF symptoms, thereby improving patient function and QoL. HCPs acknowledge that very old and frail patients seldom reach target doses identified in clinical trials. Therefore, they recommend initiating GDMT at lower doses and up-titrating very slowly, in smaller increments and wider intervals. This approach gives individuals more time to adjust to a single agent, one change at a time, and with close monitoring (Table 3, quote 8). However, the underuse of GDMT in frail older people was commonly described by HCPs. This lack of GDMT can be attributed to limited tolerability of side effects and a shift in focus to symptomatic relief to maintain QoL, if possible (Table 3, quote 9). Participants indicated that the potential benefits and harms of using GDMT in very old people with frailty are not supported by the evidence, particularly for people with HFpEF (Table 3, quote 10).

HCPs expressed uncertainty regarding how to tailor medications, owing to the absence of frailty measures in the clinical trial data and a shift in health priorities from longevity to maintaining QoL. Consumers expressed that they experienced high anxiety from using multiple-medications, the potential for drug interactions, and adverse effects or frequent medication and/or dose changes. This was particularly challenging for individuals with cognitive impairment, dementia, or difficulty adhering to medication regimens (Table 3, quote 9). This response varied depending on patients’ attitudes and beliefs relating to medications. Although polypharmacy and its associated challenges were highly prevalent among this patient population, HCPs preferred optimizing GDMT to target clinical outcomes, and they viewed polypharmacy as an issue separate from management of HF symptoms (Table 3, quote 11).

#### Identification and management of ADEs

HCPs explained that they expect adverse effects and how they consider preventing or mitigating them during the selection, initiation, and up-titration of GDMT. HCPs indicated that they closely monitor common adverse effects (eg, postural hypotension, dizziness, worsening renal function, electrolyte imbalance), but do not probe patients for all potential side effects (Table 3, quote 12). Participants described that this monitoring was made more difficult by difficulty distinguishing whether symptoms were caused by underlying comorbidities or medications (Table 3, quotes 13 and 14]. HCPs explained that they ranked the severity of ADEs mostly based on the patients’ symptomatic response and how the ADE impacted the patient’s function or increased the risk of severe outcomes (eg, hyperkalemia, hospitalization due to falls; Table 3, quote 15). HCPs described adjusting treatment if patients raised specific concerns about symptoms, which depend on patients’ preferences to engage. But consumers reported having little knowledge of what adverse effects to expect, and they likely noticed only those that impacted their QoL or their ability to engage in normal ADLs (Table 3, quotes 16 and 17).

All participants acknowledged the importance of HF pharmacotherapy and the challenges involved in weighing the benefits of preventing decompensation and worsening HF against the potential adverse effects and the impact on their QoL. These medications were associated with adverse effects that individuals accepted and often self-managed to maintain their benefits or prompted a switch to alternative medications. These adjustments are not always manageable, and some participants expressed concern about the burden of tolerating adverse effects on the QoL of very frail older individuals with shorter life-expectancy (Table 3, quote 18).

#### ADWE considerations

Most HCPs noted that ADWEs of GDMT were rarely observed. But when ADWEs did occur, they were severe and urgent (eg, angina, decompensation), requiring immediate reinitiation of the discontinued medication. Deprescribing was seen as very dynamic, particularly during acute illness (Table 3, quote 19). The most common ADWE described by HCPs was worsening of HF symptoms requiring hospitalization. Due to the lack of evidence-based guidance and loss of potential treatment benefit, HCPs expressed hesitancy to deprescribe HF pharmacotherapy. HCPs viewed the loss of treatment benefit on mortality, hospitalization, and HF symptom control to be particularly harmful in patients with HFrEF. In patients with stable asymptomatic HF, HCPs have reported harm from deprescribing (Table 3, quotes 20 and 21).

All HCPs considered maintaining GDMT in HFrEF patients to be necessary, if safe to do so, and preferred deprescribing non-HF medications to reduce the patients’ drug burden.

However, some HCPs viewed deprescribing in HFpEF as appropriate due to lack of clinical trials demonstrating clear benefit (Table 3, quote 22). HCPs provided some examples of cases in which deprescribing was considered if the benefit of deprescribing outweighed the risk, such as obvious drug-related harm (eg, risk of cardiac arrest from hyperkalemia; quote 23). Permanent deprescribing also was considered when withholding medications improved patient stability after repeated episodes of adverse events or when the treatment was no longer effective (eg, diuretic resistance, urosepsis). Most commonly, the deprescribing of HF medications was viewed as a response to progressive deterioration, end-of-life, or improved healthy lifestyle habits (eg, diet, exercise) that reduce the need for symptom control (Table 3, quote 24).

#### Facilitators and barriers to patient education about benefits and harms

Communication with older people with frailty and their caregivers can be challenging when discussing the balance between taking HF medications and maintaining an acceptable QoL (Table 3, quotes 25 and 26). Participants suggest repeating the benefits and harms of HF pharmacotherapy, referencing patients’ function, and adjusting titration regimens or drug combinations to support individuals’ QoL (Table 3, quote 27). Conversely, consumers expressed frustration regarding the lack of knowledge, confidence, and availability of HCPs to help them manage their ADEs and ADWEs from HF pharmacotherapy (Table 3, quote 28). HCPs describe having limited time and opportunity with older HF patients, particularly those with frailty, to discern the most relevant information to communicate effectively (Table 3, quote 29). These patients may require more time and support, even for minor adjustments to their routine care.

### Theme 3: access to healthcare services

#### Integrated and interprofessional coordination

Participants indicated that older people with frailty receive care across multiple health settings, which requires coordination and communication across many specialties to manage care and adverse effects (Table 4, quote 30). Although all HCPs agree that they always consider the overall patient status during shared decision-making, those in specific specialties express conflicting approaches in managing outcomes for single-organ diseases, compared to global patient function (Table 4, quote 31). This tension can make the sharing of information and responsibility for a patient’s overall care challenging (Table 4, quote 32). HCPs and consumers expressed frustration regarding the provision of ongoing care and follow-up care, particularly relating to the sharing of information and missing documentation (Table 4, quote 33). Often, the specialists who are optimizing GDMT are not the same as those who are managing adverse effects caused by the GDMT and/or treating comorbidities for individuals living in the community. HCPs expressed the need for improved multidisciplinary coordination to provide advice on how to adjust dosing for complex older patients with frailty (Table 4, quote 34). Support is needed to coordinate GDMT optimization and manage ADEs across different HCPs and healthcare settings (Table 4, quote 35).

#### Patient access to care

Participants expressed the need for frequent monitoring and access to caregiver support and healthcare services to help them adhere to HCP recommendations. Interventions involving multidisciplinary teams can improve outcomes in patients with frailty. These programs often are available after acute admission for HF to cardiology services in urban centres, but they can increase physical function, improve sarcopenia, optimize HF medications, and improve overall prognosis (Table 4, quote 36). Participants particularly value health services that involve multidisciplinary HCPs and extend into the community, so that individuals can receive support in managing and coordinating their care from home (Table 4, quote 37). However, access to frailty and HF management programs can be challenging, particularly for those in rural areas with limited access to HCPs (Table 4, quote 38). The physical and cognitive demands of coordinating access to healthcare services can be challenging for very old people with frailty, including getting to a general practitioner’s [GP] office, pathology laboratory, pharmacy, physiotherapy, specialist clinic, or hospital (Table 4, quote 39). Accessing the services needed to achieve optimal benefit from HF pharmacotherapy or manage adverse effects may not always be feasible, practical, or affordable, thereby further exacerbating health inequities (Table 4, quote 40). Although HF management programs are highly supportive, they require coordination and accessibility that may not be available to all people living in the community with frailty.

### A conceptual model to characterize frailty-related outcomes and adverse events in older people with HF

**Figure 1:**
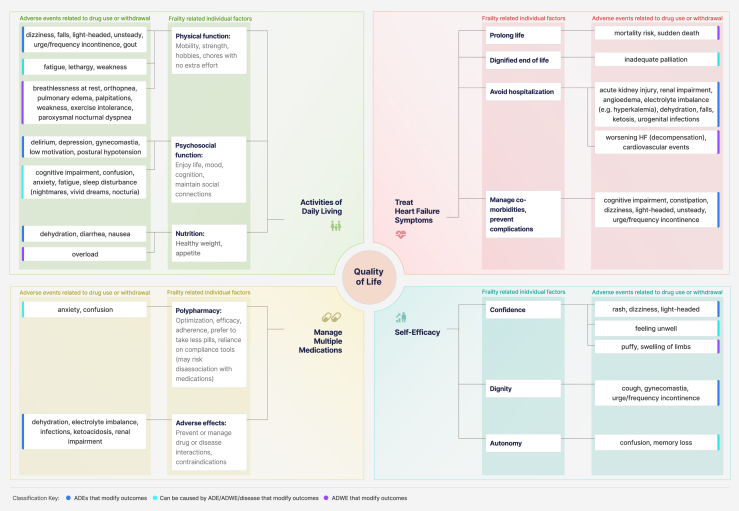
A conceptual model to characterize frailty-related outcomes and adverse events in older people with heart failure (HF). This conceptual model illustrates the relationships between the perceived effect of adverse drug events (ADEs) and adverse drug withdrawal effects (ADWEs) from HF pharmacotherapy on frailty and quality of life in older adults. The figure depicts the following 4 key domains representing goals of care in this study: activities of daily living (**green**), treat HF symptoms (**red**), manage multiple medications (**yellow**), and self-efficacy (**blue**). This dynamic diagram illustrates frailty-related individual factors as individual factors (theme 1; the first branch out from the key domain) and medication-related adverse events related to drug use or withdrawal (theme 2 ; second branch). Each domain has frailty-related factors leading to multiple levels of medication-related adverse events to consider. Frailty-related factors and adverse events can occur simultaneously and overlap across multiple key domains; thus, adverse events (eg, anxiety, confusion, dizziness, incontinence) can repeat across domains. The colours in the classification key correspond with the panel of each adverse event to indicate an ADE (**dark blue**), an ADWE (**purple**) or overlapping HF symptom/ADE/ADWE (**turquoise**) that can potentially modify frailty-related outcomes, relative to the goal of care in the key domain.

The preliminary conceptual model shown in Figure 1 illustrates frailty-related features (theme 1—individual factors) and adverse events related to medication use or withdrawal (theme 2—medications). Factors related to theme 3—access to healthcare services—not represented here, are considered potential modifiers of theme 1 and theme 2 via their action as facilitators or barriers. The interplay among aspects of frailty and commonly reported ADEs or ADWEs from HF pharmacotherapy can occur simultaneously and overlap across multiple key domains. ADWEs were most often recognized as a return of disease, in this case, HF symptoms (potentially leading to hospitalization if severe) rather than physiological withdrawal symptoms of HF pharmacotherapy. However, the same medications that are tolerated and effective in managing HF can mitigate the underlying disease, which can improve HF symptoms and frailty-related outcomes within the same domains.

Figure 1 is a dynamic diagram that can be read from the centre outward, starting with targeting goals of care in the key domain, to optimize frailty and consider potential adverse effects of treatments. Conversely, if a specific adverse event is of concern, the diagram may be read starting from the outside branch and moving toward the key domain that is potentially impacted. Examples of how this model can be used to interpret an outcome can apply to patients who want to maintain their ADLs, with physical function adequate to be mobile and strong enough to perform hobbies they enjoy and chores without excessive effort. However, medications can cause dizziness, unsteadiness, or incontinence that could lead to increased fall risks that would impact their physical function and confidence. Similarly, light-headedness can be managed by going slowly and holding onto assistive devices during sitting to standing to support ADLs. But needing more time to get up and appearing to struggle in public may feel embarrassing and lead to less social engagement and perceived self-efficacy. Fatigue or weakness can limit physical function in frailty, but careful investigation is required to discern whether such symptoms are caused by the underlying disease, ADEs, or ADWEs. Cognitive impairment and confusion were identified across all domains, supporting the need for individualized approaches and careful consideration of whether to treat delirium-related ADEs or disease progression, or adjust treatment plans according to goals of care.

## Discussion

This study identified HCP and consumer perspectives unique to medication management in older people living with HF and frailty. Although previous research has evaluated the topics of deprescribing, frailty, HF, and ADEs on their own, this study explores the combined context of these topics. HCPs and consumers share similar goals for improving patient QoL, but their approaches to achieving this may differ due to dynamic and complex influences. This novel patient-centred conceptual model proposes how tailoring treatment care plans to consider frailty status can mitigate potential adverse effects during the prescribing and deprescribing of HF pharmacotherapy. This model can be expanded further to guide future research and/or customize multidisciplinary clinical tools tailored to the complex and dynamic priorities of older people with frailty among diverse populations, such as in integrating appropriate deprescribing guidance.^22^

The findings indicate that maintaining QoL is a high priority for older people with frailty, but individuals’ goals of care are overlapping and dynamic. Participants’ descriptions align with the “capability approach“—in which well-being is valued as what is practically possible for individuals to do in their daily lives.^25^ These ADLs include those that achieve self-respect, social integration, and the freedom to accomplish things patients value.^26^ Key domains of the conceptual model align with previous research prioritizing older adult-specific health outcome goals and care preferences. Similar outcomes and preferences are described by the 4Ms framework (what Matters, Medication, Mentation, and Mobility) for older adults with multimorbidity across all care settings, but this conceptual model additionally highlights HF-specific treatment and dynamic influences of frailty.^27^ Management of HF symptoms, ADEs, and ADWEs potentially contributes to both improving and worsening individuals’ QoL, particularly during periods of adjustment.

Participants’ reasons for deprescribing were aligned with deprescribing frameworks in older people with advanced or end-stage HF, dementia, severe frailty, and high-risk drug combinations.^22^ For example, deprescribing of beta-blockers to treat HFpEF^28^ with no other indication may be considered if risk of falls outweighs the potential mortality benefits or if these benefits are unlikely to be realized because of limited life-expectancy. Conversely, emerging evidence for sodium glucose cotransporter-2 inhibitors shows improved outcomes in patients with frailty and HFpEF, most prevalent among older people.^29^ People with severe frailty and advanced HF are at highest risk of poorer outcomes, further amplifying absolute baseline risks and benefits.^30^ The view that HFpEF is difficult to treat due to the lack of evidence is a reason for deprescribing that could be explored further. Although less data support management of HFpEF than HFrEF, hesitation to deprescribe was due to uncertainty of evidence to predict recurrence of HF symptoms. Participants were less concerned with the lack of evidence on how to taper medications. An observational study showed that during admission to general, geriatric, or rehabilitation services, 60% of patients (*n* = 45) aged > 75 years with a high frailty risk and HF were deprescribed angiotensin-converting enzyme inhibitors (ACEIs) or angiotensin receptor blockers (ARBs).^31^ But deprescribing guidance, such as the screening tool of older persons prescriptions in Frail adults with limited life expectancy (STOPP-Frail) criteria,^32^ does not specifically address HF treatments. STOPP-Frail reports that physiological withdrawal effects are unlikely in deprescribing ACEIs and/or ARBs to prevent diabetic nephropathy. Clinical trials studying deprescribing are required to inform whether this applies to HF and if this precludes the long-term benefits.^15^ Future research could explore how the conceptual model could be used to support determination of what constitutes optimal prescribing in older adults with frailty and HF.

In HF, frailty associated with age-related deficiencies may reduce individuals’ ability to adjust to cardio-specific pharmacologic interventions, attenuate adverse events, or interact with other complex health conditions or medications.^33^ Multi-domain interventions (eg, physical exercise, nutritional supplements, medication review, nurse and/or allied-health support) potentially can lead to complete, prolonged, or incomplete recovery from injury or insult, but frailty may be irreversible for some individuals.^34^^,^^35^ Signs associated with frailty can also occur in single-organ failure states such as HF. A cardiac transplantation can reverse signs of frailty attributed to cardiac function, such as fatigue, slow walking speed, and muscle loss.^36^ But the post-transplantation mortality risk is higher in patients with baseline frailty compared to robust patients.^37^

In some instances, optimization of single-organ or disease-specific medications can conflict with other treatment guidelines or global function, such as acute kidney injury in acute and chronic HF. Frail patients with chronic kidney disease and HF report having poorer QoL and physical function.^38^ Contraindications or dose adjustments associated with changes in serum creatinine and decompensation can create challenges when communicating across multidisciplinary teams and can contradict patient education. Assessment of dynamic renal function and electrolyte balance require careful interpretation of serum creatinine during ACEI and/or ARB titration in chronic HF and assessment of tubular function (diuretic response) in acute HF, but they require adequate access to health services to support appropriate assessments.^39^ Efficacy and safety data from clinical trials involving older patients with frailty, real-world observational data on HF treatment, and outcomes that objectively measure frailty are needed.^40^ Guidelines on managing ADEs that interact with global patient function and/or other organ systems are needed also.

The widespread uncertainty surrounding decisions to manage ADEs and ADWEs associated with HF pharmacotherapy in older people with frailty warrants the gathering of more evidence and development of practice models to support care for this population. Older people with frailty have the right to equal access to life-saving HF pharmacotherapy. Consumer and HCP perspectives support the perceived need for evidence relating to treatment efficacy for people aged > 75 years with frailty, concerns about medication harm, equitable access to treatment and monitoring, and upholding the right to self-determination and/or caregiver decisions in individuals with cognitive impairment.^41^ Individualizing HF management that considers the dynamic interplay of ADEs, ADWEs, and frailty according to patient-centred outcomes is consistent with practical tips to adjust pharmacotherapy in advanced HF,^42^ domain management of HF,^43^ and barriers to deprescribing cardiovascular medications in geriatric patients.^44^ This study aligns with a call for more country-specific and international research to optimize prescribing in older people with functional challenges.^40^ This alignment underpins the need for further development of the person-centred conceptual model to determine how frailty risks and medication reviews can be integrated into HF treatment algorithms, tailored to specific drug classes and/or combinations, and/or adapted for multidisciplinary clinical tools.^29^ Future development and utility of this preliminary conceptual model and these decision support tools should separately target different clinicians and consumers.

This study of HCP and consumer perspectives provides new insights that consider how the frailty status of older adults can impact the communication and evaluation of ADEs and ADWEs associated with HF pharmacotherapy. Previous deprescribing literature on cardiovascular medications focuses on cardio-metabolic preventative medications, whereas treatment of symptomatic HF may prevent death and readmission while influencing frailty and symptoms.^45^^,^^46^ Medications used to treat symptomatic HF also can cause drug-drug or drug-disease interactions among older people with complex comorbidities and multi-domain frailty. Findings reflect the dynamic challenges, complexities, and uncertainties older people experience in the management of symptomatic HF and frailty. Compared to the responses in the pilot, HCPs and consumer participants struggled to discern whether adverse effects were caused by medications, progression of HF, frailty, or the effect of another comorbidity. The model shows that what individuals consider an acceptable QoL may change due to the accumulation of various health and personal outcomes that may resolve or create challenges. Findings from a broad range of HCPs perspectives, triangulated with consumer views, show how adjustments to medication management can be tailored to aspects of frailty and HF in older individuals. Interview questions were piloted, and data analysis involved 3 independent researchers and was reviewed by a multidisciplinary team. Themes were triangulated with the “capability approach” and were reviewed by multiple specialists. Common adverse effects identified for each therapeutic class in Supplemental Table S2 could be evaluated in future clinical trials.

Sampling bias was a limitation of this study. The small sample size of PWLEs and caregivers in this study reflects the challenges in recruiting older people with HF and frailty living in the broader community, including people in rural areas who are not recruited from a hospital or cardiac program. The distribution of electronic advertisements recruited fewer participants than the number in previous studies that screened and recruited patients from hospitals or cardiac clinics, often in urban centres.^13^^,^^47^ Ineligible PWLEs who did not identify as currently frail resided in urban centres with access to rehabilitation and cardiac clinics. This finding reinforces the significance of cardiac rehabilitation programs postdischarge and the need for outreach to the wider community via expanded coordination and allied health support. Interviews with PWLEs, of approximately 1 hour, were longer than those in the pilot. The extended time gave participants the opportunity to respond comfortably, to reflect on and describe their responses in relation to their circumstances, to provide rich personal stories, and to pause if the recall of events was challenging. Participants responded according to their personal experiences receiving care, with no formal assessment of whether the information they received was appropriate or comprehensive. Their experiences demonstrate the variability in education and co-management of symptoms and disease progression in usual care received from different health settings and HCPs. Future research may consider how to mitigate the burden for PWLEs while recruiting caregivers through expanded frailty and HF networks.

Frailty was not assessed objectively but rather based on self-reported perceptions from participants, which may have resulted in underestimation of frailty. HCP and consumer understanding and perception of frailty may have influenced the results. HF phenotypes were not identified, but similar QoL goals were reported for symptomatic HFrEF and HFpEF.^48^ The generalizability of the findings needs to be confirmed with future studies that include individuals from other countries, health settings, and diverse populations. The views of GPs, physiotherapists, and other allied HCPs were not represented, despite invitations to participate in this study being given to these providers. These HCPs should be considered in future research, particularly GPs, who provide more frequent longitudinal care to patients with HF, compared to cardiology specialists and geriatricians. Further development of this conceptual model could be adapted to clinical research to support the person-centred communication and monitoring of ADEs, ADWEs, HF symptoms, and frailty status with patients, caregivers, and HCPs. Further development and testing of the preliminary conceptual model may consider objective frailty assessment and recruitment from urban and rural GP clinics or frailty programs.

This study explores HCP and consumer perspectives to develop a preliminary conceptual model that illustrates how information on ADEs and ADWEs may be used to tailor HF pharmacotherapy in relation to QoL for older people with frailty. This study may guide discussions about the benefits and harms of prescribing and deprescribing HF pharmacotherapy to support patients’ goals of care. Given the absence of clinical trial data to guide treatment in older people with frailty, and the complex nature of these decisions, this research supports an individualized approach to framing HF management, ADEs, and ADWEs related to patient-centred outcomes. This study introduces a paradigm shift from pharmacovigilance reporting—which focuses on HCP responses to adverse events—to characterizing whether ADEs and ADWEs impact attributes of an individual’s well-being and ability to maintain an acceptable QoL. Evaluation of common ADEs and ADWEs identified in this study can be included in future clinical trials involving older participants with frailty. Further research is needed to determine how this information can be applied to support individualized care plans and sustainable models of care.
