# Supplementary material for: Adverse Drug Events Associated with Optimizing Heart Failure Pharmacotherapy in Older Adults with Frailty: A Qualitative Study
Source: CJC Open. 2025 Jul 2;7(10):1301–13. doi: 10.1016/j.cjco.2025.06.019 (PMC12572894; doi:10.1016/j.cjco.2025.06.019)
Supplement: Supplementary Material [file mmc1.pdf]

Supplementary Materials

Supplemental Appendix S1. Consumer and health care professional interview guides

Supplemental Table S1. Goals and clinical outcomes described by participants

Supplemental Table S2. Summary of participant reported adverse drug events by drug class

Supplemental Table S3. Summary of participant reported adverse drug withdrawal effects

Supplemental Table S4. Consolidated criteria for reporting qualitative studies (COREQ) 32-item checklist

Supplemental Table S5. Framework of emerging themes and categories

Supplemental Table S6. Additional supporting quotes of main themes and subthemes

Supplemental Appendix S1. Consumer and healthcare professional interview guides

Research Study: The classification of adverse drug reactions and adverse drug events during the optimization of heart failure medications in frail older people: a mixed-methods study

Semi-structured questions – Participant handout (consumer)

1. Please describe your experiences with frail older people with heart failure.

A: Definition of adverse drug effects

2. How do older people and/or their carers identify adverse drug effects, in general?

3. How do older people and/or their carers identify adverse drug effects from medications used to treat heart failure?

4. Which of the following types of adverse drug effects are common in frail older people taking medications for heart failure? Please describe and provide examples.
    i. dose-related (increasing/decreasing medication)
    ii. non-dose-related
    iii. dose-related and time-related (short/long term)
    iv. time-related (immediate/delayed)
    v. withdrawal (end of use)
    vi. failure of therapy

5. What are the most common adverse drug effects from heart failure medications? Which medications/drug classes are involved?

6. Which of these are the most important to patients and why?

B: Impact of adverse drug effects

7. How do older people and/or carers assess the severity of adverse drug effects and how is this measured?

8. What reasons would lead older people and/or carers to consider an adverse drug effect as mild, moderate or severe?

9. What are the most important outcomes patients and/or carers are concerned with following adverse effects from heart failure medications in frail older people?

10. How do older people and/or carers treat symptoms of adverse drug effects if:
    i. patient doesn't feel any different
    ii. stay the same (don't change over a few months, years)

iii. worsen over time

11. What kind of challenges are frail older people with heart failure faced with when experiencing adverse drug effects?

C: Causes of adverse drug effects

12. How do patients and/or carers distinguish whether adverse effects could be caused by the condition of heart failure or from a medication?

13. Are symptoms a result of taking many medications? Or a high dose of one medication?

14. Do older people usually know why they're taking the drug(s) related to the adverse effect?

D: Management

15. How do frail older people and/or carers feel about taking many medications to treat heart failure?

16. How do frail older people and/or carers assess the ongoing benefit of HF medications?

17. How do patients and/or carers weigh the harm of adverse effects with the benefits of the medication?

18. In what situations would patients and/or carers approach their healthcare provider to discuss changes to their heart failure medications?

19. Where are adverse drug effects managed (e.g. home, health care setting) and by whom?

20. What are the key considerations around reducing the dose or stopping a medication used to treat heart failure?

Research Study: The classification of adverse drug reactions and adverse drug events during the optimization of heart failure medications in frail older people: a mixed-methods study

Semi-structured questions - Participant handout (clinicians)

1. How do you manage frail older people with heart failure (HF) in your practice?

A: Definition of adverse drug effects

2. How do you identify adverse drug reactions (ADRs) and adverse drug events (ADEs), in general?

3. How do you identify ADRs and ADEs from heart failure medications, including guideline directed medication therapy (GDMT)?

4. How can ADRs and ADEs be measured?

5. Which of the following types of ADRs are commonly reported in frail older people during optimization of heart failure medications? Please describe and provide examples.
    i. dose-related (up/down-titration)
    ii. non-dose-related
    iii. dose-related and time-related (acute/chronic)
    iv. time-related (immediate/delayed)
    v. withdrawal (end of use)
    vi. failure of therapy

6. In your practice, what are the most commonly reported ADRs and ADEs from heart failure medications?

7. Which of these do you see as most important and why?

8. How do patients and caregivers report ADRs and ADEs from heart failure medications?

B: Impact (severity) of adverse drug effects

9. How do you assess/measure the severity of ADRs and ADEs?

10. How would you manage the ADR and ADE if classified as mild, moderate or severe?

11. How do you treat symptoms of ADR and ADEs that are:
    i. non-existent
    ii. stable
    iii. progressive

12. What are the factors (barriers and facilitators) that contribute to the complexities associated with ADRs and ADEs in HF patients?

13. What are the most important clinical outcomes in patients following ADR and ADE from heart failure medications? (e.g. mortality, hospitalization, HF symptoms, QoL, ADLs)

C: Cause of adverse drug effects

14. How do you distinguish whether adverse effects could be caused by progressive heart failure, underlying conditions or medication related?

15. Are symptoms a result of cascade prescribing?

16. Are symptoms due to significant toxicity?

17. Do the drugs related to the ADR have a clear indication? Are there any circumstances that this indication does not apply?

18. What are contraindications that you consider when managing patients on heart failure medications?

D: Management

19. How do you assess the ongoing benefit of heart failure medications?

20. How do you assess the harm of adverse effects with the benefits of the medication?

21. When managing medications, do you consider:
    a. preventative drugs in any/some/all circumstances?
    b. If the potential benefit may be realized because of limited life expectancy?

22. Where are ADRs and ADEs managed (e.g. home, health care setting) and by whom?

23. What are the key considerations around down-titrating a GDMT?
    a. How can we determine if down-titration of a HF medication is appropriate?
    b. What are the situations where down-titration could be appropriate to consider?
    c. What would facilitate down-titration or discontinuation of a HF medication?
    d. What are the barriers and potential risks associated with down-titration?
    e. What are the situations where down-titration should NOT be considered?

Supplemental Table S1. Goals and clinical outcomes described by participants

Consumer	Cardiologist	Doctor (geriatrician, pharmacologist)	Nurse	Pharmacist
Quality of life (QoL)<br>Do normal activities (ADLs), e.g., going to the shops<br>Joy<br>hobbies<br>social connections<br>Self-efficacy<br>Wellness<br>Dignity<br>confidence<br>autonomy<br>sense of purpose or contribution to society,<br>physically fitter,<br>being informed about decisions	Maintain or improve Quality of life<br>ADLs<br>go out and do normal things<br>Improve/maintain function, mobility<br>maintain physical exercise, rehab, muscle strength<br>improve frailty<br>enjoy living life<br>participate in their hobbies<br>social interactions<br>maintain mood (anxiety, depression)<br>maintain memory<br>good diet<br>improve patient confidence<br>live in a state acceptable to that person and family	Maintain QoL<br>ADLs<br>able to do their normal daily routine<br>frailty status<br>prevent falls<br>changing baseline function<br>maintain social connections and interactions<br>cognitive impairment<br>support patient independence and self-management	Quality of Life<br>ADLs, Function<br>support mental health (anxiety, depression)<br>support changes in cognitive function<br>maintain physical function, mobility, strength<br>weight, diet and fluid management<br>independently and comfortably perform normal daily routines (personal tasks, chores)<br>maintain social interactions/activities<br>maintain or improve sleep<br>Support patient autonomy<br>support self-management at home<br>coordinate access to health services	Improve / maximize QoL<br>recover or maintain ADLs<br>Social connections and engagement<br>emotional well being<br>maximize function (dexterity, disability, mobility)<br>balance prolonging life with living a good life<br>support patient autonomy and preference<br>patient live independently in the community
Healthy weight/appetite<br>Take less pills<br>manage side effects	manage comorbidities<br>prevent other complications (e.g., falls)<br>optimize evidence based medications<br>minimize side effects	medication management<br>optimize medications for symptom management<br>manage adverse drug events<br>patient compliance/adherence<br>reduce medication burden<br>deprescribe if appropriate<br>Individualize and adjust treatment	Manage comorbidities<br>prevent/minimize other complications (falls)<br>Support patient compliance/adherence<br>medication optimization<br>support management of adverse effects of disease/drugs<br>manage medication burden/ polypharmacy	accommodate change in cognition<br>manage weight/appetite<br>medication management<br>optimization of evidence based medications<br>prevent/manage adverse drug effects<br>patient compliance/adherence

				manage drug interactions
**Consumer**	**Cardiologist**	**Doctor (geriatrician, pharmacologist)**	**Nurse**	**Pharmacist**
Treat symptoms<br>breathless when walking<br>feet swell<br>feet oedema and walking<br>Pain free, eat what you want<br>Do chores with no extra effort<br>Prolong life, essential life saving<br>Prevent CV event (AF, stroke)<br>Avoid hospitalization<br>Dignified end of life	Symptom management/benefit<br>prevent decompensation<br>slow cognitive decline<br>Slow disease progression (improve prognosis)<br>longevity/prevent premature mortality (depends on patients)<br>reduce hospitalization<br>reduce morbidity<br>reverse remodeling, improve ejection fraction<br>Reduce risk of CV event (MI outcomes of single organ systems	Symptomatic management<br>consider global outcomes not just disease progression<br>Reduce mortality<br>adapt treatment to life expectancy<br>prevent re-hospitalization<br>support palliative/end of life care<br>manage comorbidities<br>support mental health (mood, depression, anxiety)<br>maintain physical function<br>measure and manage frailty	Symptomatic management<br>adjustments to changing functional status<br>reduce morbidity<br>Increase length of life, keep people alive longer<br>slow disease progression<br>prevent re-hospitalization<br>Support advanced care planning needs (end of life care)	Symptom management (feel better)<br>stabilize patient<br>prevent future complications<br>slow disease progression (improve prognosis)<br>reduce mortality<br>reduce hospitalization/re-hospitalization<br>improve ejection fraction<br>manage comorbidities<br>improve morbidity
Abbreviations: QoL Quality of Life; ADL Activities of Daily Living; CV cardiovascular; AF atrial fibrillation; MI myocardial infarction				

Supplemental Table S2. Summary of participant reported adverse drug events by drug class

Drug Class	Consumer	Cardiologist	Physician (geriatrician, clinical pharmacologist)	Pharmacist	Nurse
**RASI: ACEI/ARB/ARNI**	-	decrease renal function<br>angioedema	renal impairment<br>acute kidney injury (AKI)<br>hypotensive (ARNI)	renal impairment, reduced renal function<br>acute kidney injury<br>postural hypotension (high in ARNI)<br>cough (ACEI)<br>electrolyte imbalance<br>diarrhea (ARB)	renal function goes off, impaired renal function (>30% drop in eGFR, flag if 20%)<br>fry the kidneys<br>low blood pressure<br>dizziness<br>cough (ACEI)<br>increased frequency of urination (no UTI)<br>fatigue, lethargic, tiredness, loss of mobility<br>brain fog (ARB)
**Beta blocker**	-	fatigue<br>feel washed- out<br>nightmares	impotence<br>depression<br>swelling of the ankles (titration too fast)	erectile dysfunction/impotence<br>mood (depression)<br>confusion (fuzziness in the head)<br>vivid dreams/ nightmares<br>tired/fatigue/slow down<br>dizziness<br>hypotension<br>slower heart rate<br>worsen breathing, shortness of breathless (rare)<br>worsen circulatory disorders	sexual dysfunction<br>brain fog, not clear minded, don't feel like themselves<br>vivid dreams<br>falls<br>fatigue<br>constipation<br>bradycardia<br>tachycardia (underdosing)

Drug Class	Consumer	Cardiologist	Physician (geriatrician, clinical pharmacologist)	Pharmacist	Nurse
**MRA (spironolactone)**	male breasts	gynecomastia<br>sore nipples	gynecomastia<br>hyperkalemia (can cause sudden death)	gynecomastia<br>hyperkalemia<br>nausea<br>dehydration	man boobs (gynecomastia)<br>electrolytes<br>potassium goes up, hyperkalemia<br>cardiac arrest
**SGLT2-inhibitors**	-	UTI<br>urosepsis<br>euglycemic ketoacidosis,<br>increase ketones	UTI and urinary symptoms<br>diabetic ketoacidosis	UTIs<br>urogenital infections	UTI<br>vaginal or perineal issues<br>ketosis
**Diuretics (furosemide)**	need to go to the toilet, incontinence<br>risk of dehydration<br>Electrolytes<br>potassium<br>sodium<br>dizzy<br>confusion<br>trouble with memory	urinate frequently<br>confusion and trouble with memory	frequency and urge incontinence<br>sleep disturbance (getting up to go to the toilet)<br>dehydration (very dry)<br>dry mouth<br>dysuria<br>thirst<br>electrolyte abnormalities<br>low potassium<br>low sodium<br>hypotensive<br>dizzy<br>feeling weak	incontinence (urge/frequency)<br>diuresis<br>dehydration<br>hypotension<br>falls<br>electrolyte imbalance<br>hypokalemia, low sodium<br>low magnesium, low calcium,	running to the toilet, makes them pee/wee a ton,<br>frequency/urgency of urination<br>incontinence, wetting their pants, weeing all through the night, get up at night<br>dehydration<br>dry out, dry as a crisp<br>constipation<br>dry mouth, licking the top of their mouth<br>dry, scaly skin<br>really tight legs<br>low blood pressure<br>dizziness, light-headedness<br>foggy feeling
**Digitalis Glycoside (digoxin)**	-	digoxin toxicity	-	flickering in peripheral vision	-

Drug Class	Consumer	Cardiologist	Physician (geriatrician, clinical pharmacologist)	Pharmacist	Nurse
**Calcium Channel Blockers**	angioedema	angioedema<br>peripheral oedema	-	ankle oedema<br>vivid dreams<br>dizziness<br>hypotension	-
**Other**	-	-	-	-	-
**Vasodilators**	-	-	-	-	-
**Hydralazine**	-	-	autoimmune function	-	-
**Ivabradine**	-	-	-	-	sinus rhythm (bradycardia, sinus arrest)
**Inotrope (dobutamine)**	-	-	-	-	hypotension<br>dizziness

Abbreviations: RASI Renin-Angiotensin System Inhibitor; ACEI Angiotensin-Converting Enzyme Inhibitor; ARB Angiotensin Receptor antagonist/Blocker; ARNI Angiotensin Receptor-Neprilysin Inhibitor; SGLT2-i Sodium-Glucose coTransporter-2 Inhibitors; AKI Acute Kidney Injury; eGFR estimated Glomerular Filtration Rate; UTI Urinary Tract Infection.

10

Supplemental Table S3. Summary of participant reported adverse drug withdrawal effects

Consumer	Cardiologist	Doctor (geriatricians, pharmacologist)	Pharmacist	Nurse
**Nonspecific**<br>end your life	**Nonspecific**<br>lose mortality benefit<br>CV events<br>Angina<br>hospitalization<br>Trigger HF episode/exacerbations<br>HF Decompensation	**Nonspecific**<br>death<br>sudden cardiac death (e.g., stroke risk. MI)<br>Palliation/end of life<br>HF symptoms return<br>breathlessness, JVP, edema<br>HF exacerbation / decompensation<br>Rise in heart rate and blood pressure	**Nonspecific**<br>HF symptoms return<br>Rise in heart rate and blood pressure<br>HF exacerbation<br>decompensation	**Nonspecific**<br>Loss of medication benefits (e.g., cardiac remodeling, hospitalization)<br>Heart failure symptoms/exacerbation<br>pulmonary oedema<br>overload, decompensation<br>heart rate, palpitations<br>breathless, fatigue<br>Rise in heart rate and blood pressure<br>ADE not resolved
**Beta blocker**<br>stroke	**RASI: ACEI/ARB/ARNI**<br>lose long-term renal protective benefits	-	-	-
**Diuretics**<br>edema gets worse	-	-	**furosemide and thiazides**<br>CV event<br>decompensation<br>hospitalization	-

Abbreviations: HF heart failure; CV cardiovascular; ADE adverse drug event; RASI Renin-Angiotensin System Inhibitor; ACEI Angiotensin-Converting Enzyme Inhibitor; ARB Angiotensin Receptor Antagonist/Blocker; ARNI Angiotensin Receptor-Neprilysin Inhibitor; JVP Jugular Venous Pressure.

Supplemental Table S4. Consolidated criteria for reporting qualitative studies (COREQ) 32-item checklist

No	Item	Guide questions/description	Reported on page #
**Domain 1: Research team and reflexivity**			
**Personal Characteristics**			
1	Interviewer/facilitator	Which author/s conducted the interview or focus group?	7
2	Credentials	What were the researcher's credentials? E.g. PhD, MD	title page
3	Occupation	What was their occupation at the time of the study?	7
4	Gender	Was the researcher male or female?	title page
5	Experience and training	What experience or training did the researcher have?	7
**Relationship with participants**			
6	Relationship established	Was a relationship established prior to study commencement?	7
7	Participant knowledge of the interviewer	What did the participants know about the researcher? e.g. personal goals, reasons for doing the research	7
8	Interviewer characteristics	What characteristics were reported about the interviewer/facilitator? e.g. Bias, assumptions, reasons and interests in the research topic	7-8
**Domain 2: study design**			
**Theoretical framework**			
9	Methodological orientation and Theory	What methodological orientation was stated to underpin the study? e.g. grounded theory, discourse analysis, ethnography, phenomenology, content analysis	8
**Participant selection**			
10	Sampling	How were participants selected? e.g. purposive, convenience, consecutive, snowball	7
11	Method of approach	How were participants approached? e.g. face-to-face, telephone, mail, email	7
12	Sample size	How many participants were in the study?	9, 22 7,9
13	Non-participation	How many people refused to participate or dropped out? Reasons?	
**Setting**			
14	Setting of data collection	Where was the data collected? e.g. home, clinic, workplace	7

15	Presence of non-participants	Was anyone else present besides the participants and researchers?	NA
16	Description of sample	What are the important characteristics of the sample? e.g. demographic data, date	7, 9, 22 (Table 1)
**Data collection**			
17	Interview guide	Were questions, prompts, guides provided by the authors? Was it pilot tested?	Supplement S1
18	Repeat interviews	Were repeat interviews carried out? If yes, how many?	NA
19	Audio/visual recording	Did the research use audio or visual recording to collect the data?	7-8
20	Field notes	Were field notes made during and/or after the interview or focus group?	NA
21	Duration	What was the duration of the interviews or focus group?	7, 9, 22 (Table 1)
22	Data saturation	Was data saturation discussed?	7
23	Transcripts returned	Were transcripts returned to participants for comment and/or correction?	7
**Domain 3: analysis and findings**			
**Data analysis**			
24	Number of data coders	How many data coders coded the data?	8
25	Description of the coding tree	Did authors provide a description of the coding tree?	Supplement Table S5
26	Derivation of themes	Were themes identified in advance or derived from the data?	9, Table S5
27	Software	What software, if applicable, was used to manage the data?	9
28	Participant checking	Did participants provide feedback on the findings?	NA
**Reporting**			
29	Quotations presented	Were participant quotations presented to illustrate the themes / findings? Was each quotation identified? e.g. participant number	Table 2-4
30	Data and findings consistent	Was there consistency between the data presented and the findings?	9-17, 28 (Figure 1)
31	Clarity of major themes	Were major themes clearly presented in the findings?	9, 16, Table 2-4,S5
32	Clarity of minor themes	Is there a description of diverse cases or discussion of minor themes?	9-16, 18-23 (Table 2-4)

Supplemental Table S5. Framework of emerging themes and categories

Theme 1: Individual factors	Theme 2: Medication	Theme 3:Access to healthcare service
**A. Complex patient factors**<br>i. Comorbidities<br>ii. Changes in function<br>iii. Patient cognition<br>iv. HF phenotype<br>v. Frailty looks at the whole patient<br>a) impact of changes in a frail person<br>b) change in mood, depression, bereavement<br>c) frailty helps determine "ceiling" of care (patient threshold)<br>d) Equity to access care and benefits of treatment<br>vi. Definition of frailty is complex<br>a) frailty is a measure of function and mobility<br>vii. Health professional understanding of frailty<br>a) resistance to identifying frailty<br>b) objective frailty not measured in routine care so not used<br>c) rank/spectrum or measure severity of frailty (how frail)<br><br>**B. Supporting patients' autonomy**<br>i. Dignity<br>a) reliance on others, loss of autonomy<br>b) adjust to societal and social expectations of older people<br>c) patient confidence<br>d) importance of social connections<br>ii. Patient engagement and choice<br>iii. Patient support or advocate<br>iv. Goals of care and patient expectations<br>a) Quality of life (QoL)<br>b) activities of daily living (ADLs)<br>v. Tailored to each person<br>vi. Patient is stable<br>vii. Decisions at end of life	**A. Medication management**<br>i. Evidence based medicine (guidelines)<br>a) Prioritization of clinical outcomes vs patient<br>ii. under use of guideline directed medical therapy (GDMT) (ACEI/ARB, BB, MRA, SGLT2-I<br>a) tolerability (benefits vs risks)<br>b) focus on symptomatic relief of HF<br>iii. Polypharmacy is a separate issue from prioritizing clinical outcomes<br>iv. dynamic, when ADE resolved/stable (e.g. BP, renal fxn), rechallenge and up-titrate GDMT again<br>a) aim to stabilize the patient. Change is challenging to manage<br>b) patient prognosis and goals of therapy<br>v. adjustments and limitations of GDMT in frailty<br>a) lower doses/slow up-titration/wider intervals if tolerated/ flexible dosing<br>b) single agent vs combination/multiple agents<br>c) One change at a time<br>vi. Gap in the evidence for GDMT<br>a) Lack of tools to rank or score level of risk to optimize pharmacotherapy (e.g. modified Rankin score in thrombolysis)<br>b) identify which frailty phenotype benefit from GDMT in which HF phenotypes<br>c) Research to personalize appropriateness of GDMT according to patient outcomes and "acceptable" quality of life in frail older people<br>d) lack of evidence to treat HFpEF<br>e) absence of frailty measure in clinical trial data is challenging to draw conclusions (gap in evidence)<br>vii. drug interactions<br>iii. time frames or monitoring intervals<br>ix. patient adherence/non-compliance<br>a) medication compliance tools (pros/cons)<br>b) Medication reviews<br>c) patient given enough time to adjust to tolerable changes<br>x. patient attitudes and beliefs about medicines, health, HCPs<br>a) patient anxiety around medications<br>b) polypharmacy, high number of medicines a concern	**A. Integrated and inter-professional coordination**<br>i. Multiple health care settings<br>a) communication between HCPs<br>b) Sharing of information and decisions across specialties<br>c) leadership/responsibility of overall patients care<br>d) Differing priorities between specialties<br>e) accountability<br>ii. Monitoring and follow up<br>a) Ongoing or continuity of care<br>iii. Need coordinated multidisciplinary expertise to navigate complex frail older people<br>a) Focus on one organ vs whole patient even though considering overall patient status<br>b) Provide advice how to adjust dosing for specific ADEs between settings and health providers<br><br>**B. Patient access to care**<br>i. Challenges to accessing care<br>a) Health services and/or medication related costs<br>b) Available support to manage HF and frailty<br>ii. Co-ordination of care<br>a) Physical access or cognitive coordination

	**B. Identification and management of ADE**<br>i. Prevention of ADEs during selection and initiation of medication<br>ii. Identify, report, investigate symptoms<br>a) sources of information used to identify ADE<br>b) ADEs a concern raised by patient vs probed by HCP<br>c) symptoms not experienced prior to initiating medication<br>d) Uncertain if symptoms caused by comorbidities or medications<br>e) Validation if stop and see symptoms improve, then restart and observe if ADE returns (rechallenge confirms or maybe something else)<br>iii. Severity of ADEs<br>a) perceived impact of ADE (e.g., impact on function)<br>b) signs ADEs no longer tolerable<br>c) have sick day plans for moderate to serious ADEs<br>iv. Lack of ADE patient knowledge<br>a) unaware which medication is causing ADE<br>v. weigh benefits vs risks<br>a) ADE vs decompensation/worsening HF<br>b) accept chronic ADEs<br>c) patients manage to live with (anticipated) ADEs and make adjustments<br>d) Cascade prescribing<br>e) preference to switch to alternative medication	

	**C. Adverse drug withdrawal effects (ADWEs) considerations**<br>i. lack of evidence on deprescribing<br>a) hesitancy to deprescribe HF medications<br>b) can cause harm and worsen QoL<br>ii. weigh risks vs benefits of deprescribing<br>a) obvious harm of ADE warrants being stopped<br>b) consider patient stability following deprescribing<br>c) deprescribing a sign of worsening prognosis (treatment stops working)<br>d) deprescribe if improve healthy lifestyle, habits, exercise, diet (reduce need/benefit if not symptoms or reduced risk)<br>iii. Preference/attitudes to deprescribing<br>a) deprescribing dynamic not always permanent<br>b) preference to deprescribe non-HF medications<br>iv. Identification and monitoring of ADWEs<br>a) frequency of ADWEs<br>b) safe, no change in patient after discontinuation	
	**D. Facilitators and barriers to patient education about benefits and harms**<br>i. balance medication recommendations with QoL<br>a) Communicating about frailty and impact on treatments<br>ii. Navigating evidence of HF medications<br>iii. Communication to patients about medications and ADEs<br>a) limited time for effective communication<br>b) challenges to patient -HCP communication<br>c) Shared decision making	

Abbreviations: HF = heart failure; QoL = quality of life; ADL = activities of daily living; GDMT = guideline directed medical therapy; ACEI = angiotensin-converting enzyme inhibitor; ARB = angiotensin receptor blocker; BB = beta-blocker; MRA = mineralocorticoid receptor antagonist; SGLT2-I = sodium-glucose cotransporter-2 inhibitor; BP = blood pressure; HFpEF = heart failure with preserved ejection fraction; HFrEF = heart failure with reduced ejection fraction; HCP = healthcare professionals; ADE = adverse drug event; ADWE = adverse drug withdrawal effect.

Supplemental Table S6. Additional supporting quotes of main themes and subthemes

Theme 1: Individual factors	
1a. Complex patient factors	
Multiple complex issues to consider	*"Initially manage the frail older person, rather than isolated heart failure. The first for older frail people is to have the ceiling of care in place. In these frail people, probably the bar needs to set unfortunately a little bit lower compared to the normal healthy younger adults. If it's pre-frail, maybe there's some room for movement, and that medication might help them to become less frail." Cardiologist 27*
Defining frailty status	*"Frailty tends to indicate physical function. Under the banner of frailty, you've got dementia, cognitive impairment, and that that's a huge issue when you are prescribing medications. Often their blood pressure and their creatinine and electrolytes don't allow you to treat them with optimal medications. It's a fine line between keeping them upright and having their heart failure treated adequately. We're often treading that line in our patients." Geriatrician 19*
1b. Supporting patients	
Patient independence	*"I struggle a bit. It's navigating that without actually worrying or hating all of it. All they want to know [is if] you're getting better. Sometimes you're not feeling all good, don't feel much better. It's a tricky path we tread as we get older and with the frail." Consumer 55*
Maintaining social engagements	*"From mom and some people in aged care, having friendship, having warmth and comfort and socialization is very important. Is very important because they've left their home. They've lost all their independence. They've often lost their partner, and I think it can be very lonely. I think you can't underestimate that. Your emotional wellbeing". Consumer 54*
Support of carers and advocates	*"A lot of people don't like the feel of any vague kind of dizziness in an older person. The fear of falling is big, knowing they can really hurt themselves if they fall. And if they feel a fall, their fall can then trigger lack of confidence, and this cycle of being anxious. I can understand why people don't want to be dizzy and don't want to fall." Cardiologist 25*
Flexible and tailored for limited function and frailty.	*"Goals that we talk about, we have made ourselves as healthcare providers. But with frailty, a lot of these patients have lived a very long life. The goals for them are fundamentally different to what we have traditionally thought of as important endpoints in care." Cardiologist 28*

Theme 2: Medications	
2a. Medication management	
Consumers prioritize maintaining their QoL	*"The aim of most medications is to make it to old age, not to prolong old age. A lot of my older patients who are frail, say that when their time comes, time comes; they want to feel good until then. But a lot of people say they want to live long as they can. So it's a half-half."* Cardiologist 25<br><br>*"The fact that they can't get off the chair, or they're exhausted, or they feel they're gonna fall over every time they stand up. That would be the thing in my experience that patients would want managed."* Nurse 11
Health professionals prioritize clinical outcomes	*"There's a belief that you should practice evidence-based medicine. You should be on these medications and at the dose that's proved to be effective. So their starting point is we need to get people to those doses. That's their paradigm and there's a logic to that."* Geriatrician 23<br><br>*"The GDMT, although it's great in theory in people who are otherwise robust and have really good cardiogenic reserve, I think in elderly patients is really challenging because a lot of the guideline directive of medical therapy has side effects that are overlapping with the syndrome that they have come to hospital with."* Cardiologist 28
Optimizing GDMT entails dynamic and frequent changes	*"I find it really hard sometimes with the hospital [admissions]. We'll spend all this time in community titrating things and then they just like stop it because they're a bit hypotensive and things like that, and you're like, 'No!'."* Nurse 26
Targets in frail older people different than those from clinical trials and require adjustments.	*"We individualized that and went very slowly with him. We did half a tablet of Entresto every couple of weeks. And finally got him up to maximum dose. Even though that's not guideline, at least he's got there and he's feeling better and he's been safe in that process."* Nurse 11<br><br>*"Sometimes the reduction in frailty through the physical rehabilitation will allow the later up-titration of the medical side of things and you allow them that time for things to come together. You really see that early post-heart failure hospitalization for example."* Cardiologist 12
Under-use of GDMT in frail older people	*"The overarching treatment goal for these people is probably the symptom management rather than aiming for the full, reverse remodeling of their heart failure. Rather than choosing [all] therapy of heart failure, you might just pick a couple. That also can help with symptom management. The most recent guideline directed medical therapy, SGLT2-i and Entresto, they actually help decongesting the heart failure. They have the natriuresis side effects [which] also help with symptomatic management. In fact, Entresto actually can give people more energy and help decongestion."* Cardiologist 27

Evidence for optimization of in very old frail people	*"Our aim should be sort of more for the symptom management rather than the aim for prolongation of life. Unless of course they have a very good baseline function and things like that, then we should definitely give them that option. But otherwise it should be less, sort of be more conservative and more patient centre-focused approach." Cardiologist 27*
Absence of frailty measures in clinical trial data	*"If there is evidence that the guideline directed medical therapy we're offering patients who are already very frail, compromises their quality of life, then that's something we need to address. Because there's no point living longer in a state of being that is not acceptable to that person or their family." Cardiologist 28*
Anxiety of using multiple medications	*"I'm careful how when I take my medications. They do cause digestive problems and anxiety." Consumer 55*<br><br>*"Once a patient's got an adverse event to a medication, which may be due to an excessive dose, it's really hard to regain their confidence in it [the medication]. If you do it slowly, then build their confidence in [the medication], they're less likely to have that [adverse event] and get the anxiety that goes with it. The anxiety can cause adverse events. Six to nine months time to dose up-titrate, we will get the dose up there eventually." Cardiologist 12*
Patients' attitudes and beliefs around medicines.	*"Patients who are very good and methodical are able to manage their medications. We encourage them to maintain some control over their medications for as long as they are able, because then they have an understanding of what they're taking and why they're taking it. When it does go into the Webster-pack, they start to lose an association with what is in there and why they're on it. That can make it harder for us as clinicians sometimes to manage the changes as well and for patients to report changes." Nurse 14*<br><br>*"In a way, that's a necessary evil, but it also robbed dad of his autonomy as well. For someone like dad who had actually been managing his meds quite well, until the old Webster pack came along and did his head in." Consumer 53*<br><br>*"For those who are home alone with some degree of cognitive impairment where we don't think they'll reliably take their medications, they need to be on a once-daily regime, so that if needed, community nursing can go out and administer those medications. Otherwise, there's no point if they are taking the same medication twice and mixing up their medications. Geriatrician 20*
Challenges with polypharmacy	*"[Polypharmacy] is just not a clinical problem that needs a solution. Just figure out the solutions." Pharmacologist 24*<br><br>*"Quite often we find patients who have this great medication list, and you look at their medications and they're beautiful and you think, 'this patient's optimized.' They come into hospital and the nurses start giving them the medication as it's been prescribed, and that's nowhere near what they're taking. Five seconds later, their kidneys are terrible. That patient looks like they've got optimized." Nurse 6*

2b. Identification and management of ADEs	
Expected adverse effects	*"For older people with multiple problems, we will selectively consider nebivolol over other beta blockers because they tend to tolerate blood pressure. If they have multiple hospital admissions or multiple presentation with a lot of urine infections, probably not a good idea to add on the SGLT2-I, similar for MRAs like spironolactone. In frail people, the renal function in advanced age will not be very good."* Cardiologist 27
Monitor common adverse effects	*"[ADEs] are plentiful, frequent and common. It might be hypotension, renal impairment, or acute kidney injury. That patient may have a fall related to hypotension. Few number of patients complain of incontinence or frequency of urine. But once we've reached a euvolemic state, that settles that symptom. The beta blocker, fatigue would be really quite common, [and] less often patients will report really vivid dreams and sometimes a switch will improve that. We do still see a cough associated with the ACEI. But it's usually kidney, potassium, creatinine and hypotension and potential fall.* Nurse 14
Difficulty distinguishing whether symptoms caused by underlying comorbidities or medications.	*"It's close-up follow-up with the patients and see what they're experiencing in terms of medication side effects, which wasn't pre-existing, previous before the actual medication started. Otherwise, it's hard to stay which is which."* Cardiologist 27<br><br>*"I'm always comparing myself to what I was doing, rather than just settling into the moment and working through the bad. It's not over medication, but it's just come from all that [has occurred]."* Consumer 55
Severity of ADEs based on the patients' symptomatic response.	*"The adverse effects that I'm most interested in are things that are going to increase the risk of falls, blood pressure, and things that are going to throw the kidneys off."* Pharmacologist 24
Adjusting treatment depends on patient engagement.	*"I do find that some of the medications I take do give me digestion problems. I also feel, and I have talked with my doctor, that I get very anxious and it may also be coming from some of the medications I had to take for atrial fibrillation and enlarged heart."* Consumer 55<br><br>*"The very frail, silent generation, those born between the wars, they don't tell you anyway. It's like, "It's nothing." They don't want to be any bother."* Nurse 10
Consumers awareness of adverse effects	*"It's hard to say what's important for the patient when they don't know they're having an adverse effect."* Nurse 11<br><br>*"The patients don't really tell you that they feel dizzy, lightheaded. Especially the aging population, I don't think they actually know which one of the medications is causing it."* Cardiologist 27

Weighing the benefits and harms of preventing worsening HF with adverse effects.	*"Because of the way that it makes them urinate so frequently because of other comorbidities, primarily obesity and osteoarthritis, we've seen a lot of patients who have stopped taking medications that make them pee. They come with florid, decompensated congested cardiac failure, like floridly." Cardiologist 28*<br><br>*"You don't know whether the medications become stronger because their organ function is getting older. You don't know whether organ functions go, [and] medications have become stronger because they're not profusing and everything becomes relatively stronger because the heart's failing, Or because the heart is failing, and they can no longer keep their blood pressure up." Pharmacologist 24*<br><br>*"But [the medication will] work and most of the doctors won't go, 'okay, we'll just get rid of that one.' They'll try and find something else, another combination. But they may withdraw a drug and say, 'the side effects are worse than the benefit. So, let's try this.'" Consumer 54*<br><br>*"Can their renal function take the diuretic, do we dry them out? If it's clear they're getting symptomatic benefit, and that the cost of that is a new baseline renal function, then so be it." Geriatrician 23*
Self-manage adverse effects to maintain benefits of medications.	*"The fatigue is incredible at times, but it's improving a little bit. I've got try and manage it, with public obligations and commitments." Consumer 55*<br><br>*Patients themselves, the older ones who really understand their Lasix, will not take it on the days when they're going to do something. They self-manage the adverse event in that way. Nurse 10*
Adjustments are not always manageable	*'It depends on the spectrum of how much they're feeling worse, if they're actually worse. A lot of people tolerate medications that make them feel awful, especially in the older age. Cardiologist 25*
**2c. ADWE considerations**	
Identification of common ADWEs	*"[Patients] stop taking the medications in the hope that they can stop peeing and stop having to use the bathroom. But then, they decompensate. By that point it's too late, they need to come into hospital. That is a good example of where drug withdrawal, because of maintaining quality of life, has actually come at a cost to the patient. And I think that the answer is not drug withdrawal. It's treating the underlying problem, which is profound obesity, sarcopenia and osteoarthritis in these elderly frail patients." Cardiologist 28*
Hesitancy to deprescribe HF-medications.	*"Unless they're end of life, any therapy is beneficial to them. If your LVEF is stronger, you are less likely to have a lot of the issues that come with heart failure as a very simple way of looking at it. Unless someone you think is going to die in three months or less, then stopping heart failure therapy for no reason at all doesn't make sense to me." Cardiologist 29*

Deprescribing dynamic, particularly during acute illness.	*"As long as you can, keep titrating as much as you can, as much time as possible on the maximal optimal doses is the goal. Yes, down-titrate if required, up-titrate again when you can. Really dynamically; even the patients who've sort of entered unequivocally sort of palliative phase, terminal days, even then [I'm] very reluctant to totally eliminate the medications, unless it was for symptoms."* Cardiologist 12
Loss of treatment benefit is harmful in patients with HFrEF.	*"We're trying to balance things because you don't want to rush to remove anything that's going to be potentially beneficial to the heart function and remodeling of the myocardium. But also, you don't want to be increasing risk of fall and re-hospitalization due to that. That's not an easy question to answer as to when do I rush in to do something." Nurse 14*
Potential harm in deprescribing in asymptomatic stable patients.	*"I stopped their digoxin. 'This isn't doing anything. You've got CRT, you're on all the medications, it'll be fine.' Ten weeks later, "I'm quite short of breath." She was in terminal decline though I ramped up the hydrochlorothiazide. Her NT-proBNP went from 4,000 to 22,000. She's like, "I'm still really bad. I went to cut my fingernails and I became short of breath." What happened is that digoxin was enough to keep her heart rate down so that her CRT could do its job. When I removed the digoxin, her heart rate went above the level at which the CRT kicked in and she went back to her natural dyssynchronous rhythm. She was dioxin dependent." Pharmacologist 24*
Preference to deprescribe non-HF medications.	*"Generally, we will be trying to deprescribe. [But] especially not with heart failure specific medications, they are more sacrosanct than a lot of other medicines. We take some of those medications down and taper, hopefully one at a time. In general, heart failure meds would be the last to go. Most of the time we'd be taking other things out and they would be left in until someone was palliative." Pharmacist 4*
HFpEF has lack of clinical trials demonstrating clear benefit.	*"One other patient we started on, he has HFpEF [and] pulmonary hypertension. We thought could have been some benefit to him, but he didn't feel it made much difference to his symptoms so [we] didn't persist with it." Nurse 14*
Obvious harm warrants deprescribing.	*"Sometimes we make the decision this is too dangerous for this person. We think we should stop it completely. If someone with a massive prostate, coming with a urosepsis, generally we will probably stop it permanently. If this is their second or third episode of urosepsis with the SGLT2 inhibitor. The cost benefit actually doesn't weigh in favor of those patients because they're very close to dying every time they come in." Cardiologist 28*
Considerations for permanent deprescribing	*"If I get readmissions to the service, it'll be people with HFpEF who for one reason or another [but] usually quite different, don't respond to medications in the same way. Maybe a bit diuretic resistant. Nurse 11*<br><br>*"With both ARBs and beta blockers, people have thought, I just don't feel right. I feel like my brain's in a fog. Then you back off and then they feel better again. I think sometimes the optimal dose we see to maximum tolerated." Nurse 26*

Deprescribing a sign of progressive deterioration or end-of-life or improved lifestyle.	*"A more holistic point of view is really useful. Patients, they don't want to have gout, they do not want to have pulmonary oedema that is much more motivating than lose weight. Look after yourself exercise, they're like, 'Yeah everyone tells me that.' If you say, 'I'm going to give you less diuretics because you haven't got so much fluid on board. You can drink the bath, you're still going to be thirsty. If you drink less, you're going to feel well and you're not going to have a flareup of your gout because I don't need to give you this medication.' They're like, 'oh yeah, I'm in'. Nurse 6*
2d. Facilitators and barriers to patient education about benefits and harms	
Challenges to communication challenging when discussing balancing ADEs and QoL.	*In the acute stages coming out of the hospital, I'd say the benefit outweighs the risks of continuing with the medication, use simple words like, to keep them safe, "This medication is keeping you going, keeping you chugging along. You may be going to the bathroom a lot, but you're less breathless. You're able to walk, you're able to mobilize more. And this is a short-term thing. You might stay on these meds, but it's keeping your heart rested and so you can go for longer." Nurse 15*
Facilitating communication repeating the benefits and harms of medications, referencing patients' function and QoL.	*"We use functionality because the word frailty can have negative connotation if you are telling them they're frail. I focus on safety because you don't want anything to happen at the expense of increasing a drug that they have a fall into a hip and then they've pneumonia and in hospital." Nurse 26*<br><br>*"For the patients we see, a large proportion do not know what medications they're taking. If they can list their medications, they don't necessarily know the indication. If we were to communicate all of that information, I'm not sure how much of it they would retain." Geriatrician 20*
Limited time and opportunity for health professionals to manage ADEs and ADWEs is a barrier	*"[Nurses] spend a lot of time explaining. When they see their cardiologist and their GP, there's usually not a lot of time, they just say, 'these will help your heart sort of thing' for 10 minutes and then the [GP] get this letter from me with this list of demands and they need this and this... They're like, 'oh my god.'" Nurse 26*<br><br>*"Especially if you're in a busy pharmacy, you want to call the doctor to explain what's happening and follow up. It's quite rare for that to happen because you need [to consider] the time factor in the community setting." Pharmacist 5*
	"Generally, we will sit down with the family and instead of going through all the drugs, we'll pick the drugs that have either, been complicated this admission or have caused them to come into hospital. And we'll just lay out the information for the family and our recommendation, to get the family's point of view. Especially in the patients that have cognitive impairment or dementia." Cardiologist 28

Theme 3: Access to Healthcare Services	
**3a. Integrated and inter-professional coordination**	
Care across multiple health settings	*"True adverse effects, or the serious ones, don't come to a clinic so it's hard. You get the reason why something started was stopped taken out of context, it's tricky. There's a lot of role for heart failure specific clinics and nurse practitioner education clinics where someone is dedicated and patients are followed a lot more." Cardiologist 29*
Manage outcomes for single-organ diseases compared to global patient function.	*"[Cardiologists] can always advise on those medications, but at the end of the day, their treatment optimization decisions need to be made in conjunction with the geriatrician. Some cardiologists can be very aggressive and will say 'this treatment [is needed]' which may not necessarily be suitable for this patient. At the same time, geriatricians will sometimes push us to do certain things that we think is not appropriate for that particular age group. It's completely individualized, based on the clinician, regardless of their specialty training." Cardiologist 27*
Sharing information and responsibility of patient's care.	*"[Cardiologists] are the most influential people, and they treat the majority of people with heart failure, along with the GPs. Geriatricians, look after that anecdotal subset of people." Geriatrician 23*
Responsibility of managing adverse effects in individuals living at home.	*"I'd probably be more inclined to ensure that I check blood in patients who have really any change in regular diuretics. Any big change, any change really in ACE inhibitors or ARB or Entresto. I'd be doing a few more frequent checks for those things. A bit slower probably as well. I must admit as a cardiologist they're not seeing people that frequently anyway. Sometimes I might, see people kind of probably the most frequently three to six months; and then least frequently about every year. Timelines are a bit different in specialist land cause, you're not seeing everyone every two weeks or every month like you might in GP world." Cardiologist 25*
Need improved coordinated multidisciplinary expertise to navigate care.	*"Furosemide, is often prescribed either from hospital or by the GP as a drug that's like a get out of jail free card. 'If you're putting on weight, you've noticed your feet are getting swollen, or you're becoming a bit more breathless, just start taking some furosemide and see how you go.' Which often is an indicator that they need to seek care rather than taking furosemide. But on the flip side, a lot of patients have regular furosemide because of how poor their ejection fraction is." Cardiologist 28*<br><br>*"I've got much better at deliberately involving the cardiologist in my deprescribing. I'll send an email straight away. Whereas previously, I just deprescribe. It's not good patient care cause what'll happen is you'll reduce it, [the patient] will then go and see the cardiologist and restart it. And the poor GPs caught in between." Geriatrician 23*
**3b. Patient access to care**	
Frequent monitoring and access to health services.	*"It's the burden of the treatment. The titrating and the people coming in and out of their life. It's actually the diuretic and the pill burden and the burden of getting to and from medical appointments and things in the frail, that I think potentially has equal consequences as the tablets." Nurse 10*
Community support to coordinate care	*"In the heart failure program, I don't know whether the outreach team takes on very demented or very, very frail people. Geriatrician 20*

Access to healthcare services not always possible, further exacerbating health inequities.	*"Somebody who is maybe not so wealthy or maybe homeless, they don't have any supports. You've got to take all that into consideration of how the person's dealing with their illness and whether they can afford or take their medications." Nurse 6*
